# A New Tool for Analyses of Whole Genome Sequences Reveals Dissemination of Specific Strains of Vancomycin-Resistant *Enterococcus faecium* in a Hospital

**DOI:** 10.3389/fmed.2021.733676

**Published:** 2021-10-27

**Authors:** Lis Marbjerg, Caroline Louise Stougaard, Sophie-Amalie Grønhøj Sørensen, Amalie Vørs Thomsen, Lis Wang, Lise Andersen, Thomas Emil Andersen, Birgitte Kallipolitis, Michael Kemp

**Affiliations:** ^1^Department of Clinical Research, University of Southern Denmark, Odense, Denmark; ^2^Department of Clinical Microbiology, Odense University Hospital, Odense, Denmark; ^3^Department of Biochemistry and Molecular Biology, University of Southern Denmark, Odense, Denmark; ^4^Department of Regional Health Research, University of Southern Denmark, Odense, Denmark; ^5^Regional Department of Clinical Microbiology, Zealand University Hospital, Slagelse, Denmark

**Keywords:** vancomycin resistant enterococci, whole genome sequencing, bioinformatics, genomic epidemiology, infection control, surveillance

## Abstract

A new easy-to-use online bioinformatic tool analyzing whole genome sequences of healthcare associated bacteria was used by a local infection control unit to retrospectively map genetic relationship of isolates of *E. faecium* carrying resistance genes to vancomycin in a hospital. Three clusters of isolates were detected over a period of 5 years, suggesting transmission between patients. Individual relatedness between isolates within each cluster was established by SNP analyses provided by the system. Genetic antimicrobial resistance mechanisms to antibiotics other than vancomycin were identified. The results suggest that the system is suited for hospital surveillance of *E. faecium* carrying resistance genes to vancomycin in settings with access to next Generation Sequencing without bioinformatic expertise for interpretation of the genome sequences.

## Introduction

Surveillance of specific microorganisms in hospitals and evaluation of genetic relationship of isolates is fundamental in detecting and interrupting transmission. Whole Genome Sequencing (WGS) offers a unique tool for analyzing relationship of vancomycin resistant enterococci (1–3), and may serve as a method for typing the bacteria for local surveillance. While bioinformatic analyses of WGS data have generally been hampered by complicated procedures, tools requiring no bioinformatic skills are now available to support infection control. Here, a local infection control unit retrospectively analyzed all available genome sequences of *Enterococcus faecium* carrying resistance genes to vancomycin isolated at a hospital from 2014 to 2018 by a new online tool, 1928 Diagnostics. Based on WGS data 1928 Diagnostics analyze core genome genetic relationship and genetic antibiotic resistance mechanisms in individual bacterial isolates (4).

## Materials and Methods

### Clinical Isolates

The study was carried out at Odense University Hospital, which is a >1,000 bed university hospital placed at two geographical locations. Isolates of *E. faecium* carrying *vanA* and/or *vanB* gene from clinical (non-screening) samples were analyzed. The first isolate from each patient in the years 2014 to 2018 was included. Date and place of sampling was recorded. No patient information was included. Core genome Multi Locus Sequencing Typing (cgMLST) of some of the included isolates have previously been reported (5, 6).

### Whole Genome Sequencing Data Analyses

WGS was carried out by 2 ×150 bp paired end sequencing using a MiSeq instrument (Illumina, San Diego, CA, USA). Compressed unassembled sequence files were uploaded to the 1,928 Diagnostics pipeline (https://www.1928diagnostics.com/). The system initially performed a quality check based on sequencing depth/coverage. Results for individual isolates processed by 1,928 Diagnostics were presented with MLST sequence type (ST) and antibiotic resistance gene profiles. A cgMLST based Unweighted Pair Group Method with Arithmetic mean (UPGMA) dendrogram showing number of allelic differences was generated by the system. One thousand nine hundred twenty-eight Diagnostics uses the resqu database for identification of horizontally transferred antibiotic resistance genes (https://www.1928diagnostics.com/resdb/).

Isolates with 20 or less allelic differences were considered genetically related (7). Clusters of more than three genetically related isolates were studied in detail by the single nucleotide polymorphism (SNP) analysis function in 1928 Diagnostics. Closest Genbank complete chromosome sequence, identified by KmerResistance (https://cge.cbs.dtu.dk/services/kmerresistance/) using sequence files from a randomly selected isolate from each cluster, served as reference. The exclusion distance was set to 10.

## Results

### MLST

A total of 64 genomes from *E. faecium* were available for analyses.

**Figure 1:**
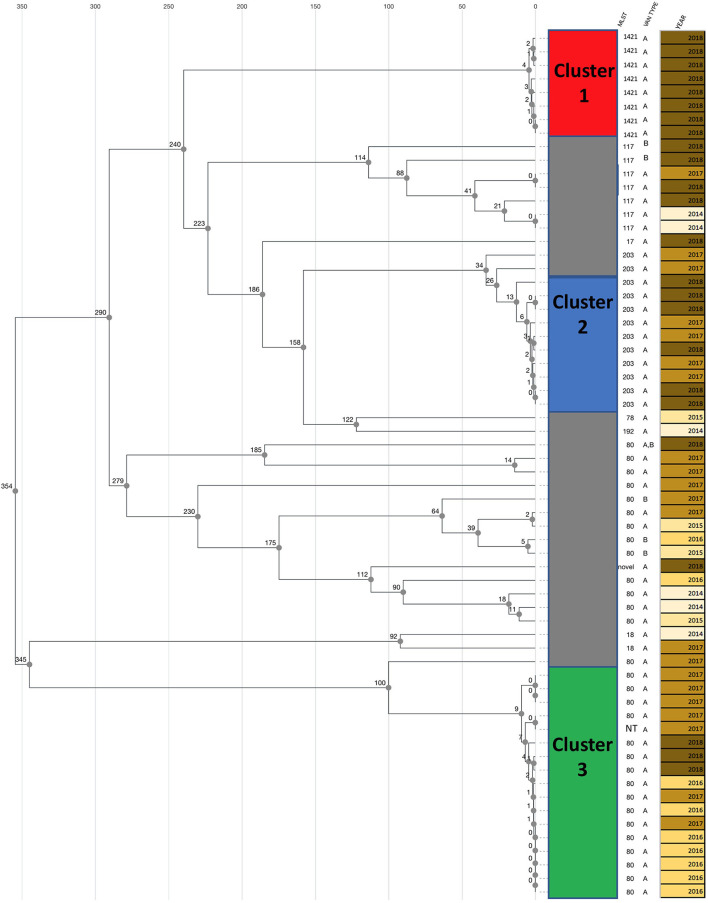
cgMLST based UPGMA clustering dendrogram of clinical (non-screening) *E. faecium* carrying resistance genes to vancomycin isolated 2014–2018 isolated at a large university hospital. Three clusters of genetically related isolates were identified. The number of allelic differences between individual isolates are indicated at each node. The MLST sequence type and the van gene (*vanA* or *vanB* gene) is indicated for each isolate next to the dendrogram. One isolate was not typable (NT). One isolate carried both *vanA* and *vanB* gene. Year of isolation is indicated for individual isolates.

Fifty-eight isolates carried the *vanA* gene, five carried *vanB* gene and one isolate from December 2018 carried both *vanA* and *vanB* gene (Figure 1). One isolate was not typeable by MLST. This was confirmed by MLST-2.0 (https://cge.cbs.dtu.dk/services/MLST/), which failed to detect hits in the pstS locus. The most common MLST type was ST80, including 30 isolates of which 26 carried *vanA* gene, three carried *vanB* gene, and the one isolate carrying both *vanA* and *vanB* genes. The UPGMA dendrogram showed large allelic variation in the core genome of the ST80 isolates (Figure 1). Twelve isolates were ST203 and eight were ST1421.

### Core Genome MLST, Epidemiology, and Antibiotic Resistance Gene Profiles

**Figure 2:**
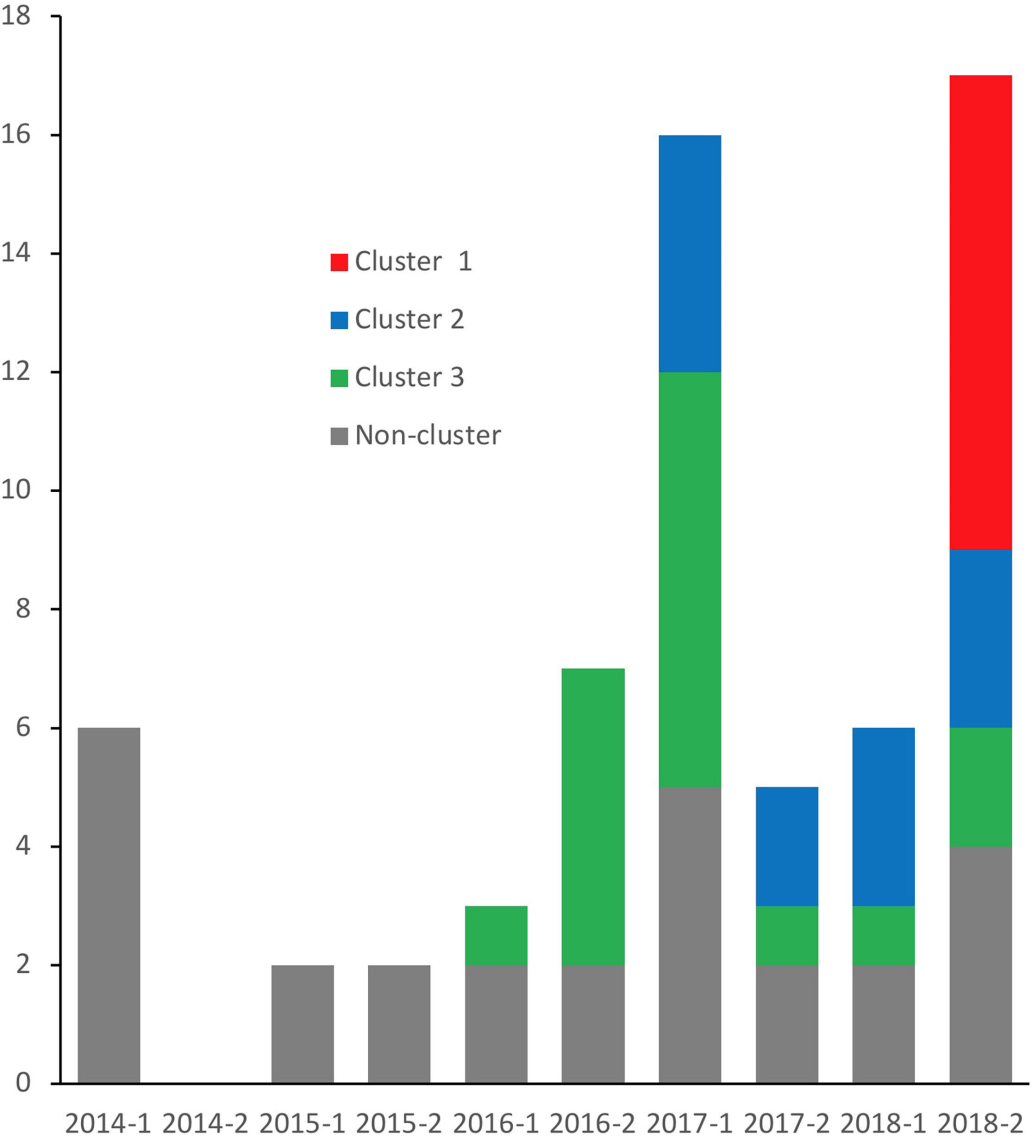
Number of isolates per half a year. Isolates of the three clusters identified from Figure 1 are indicated by color.

**Figure 3:**
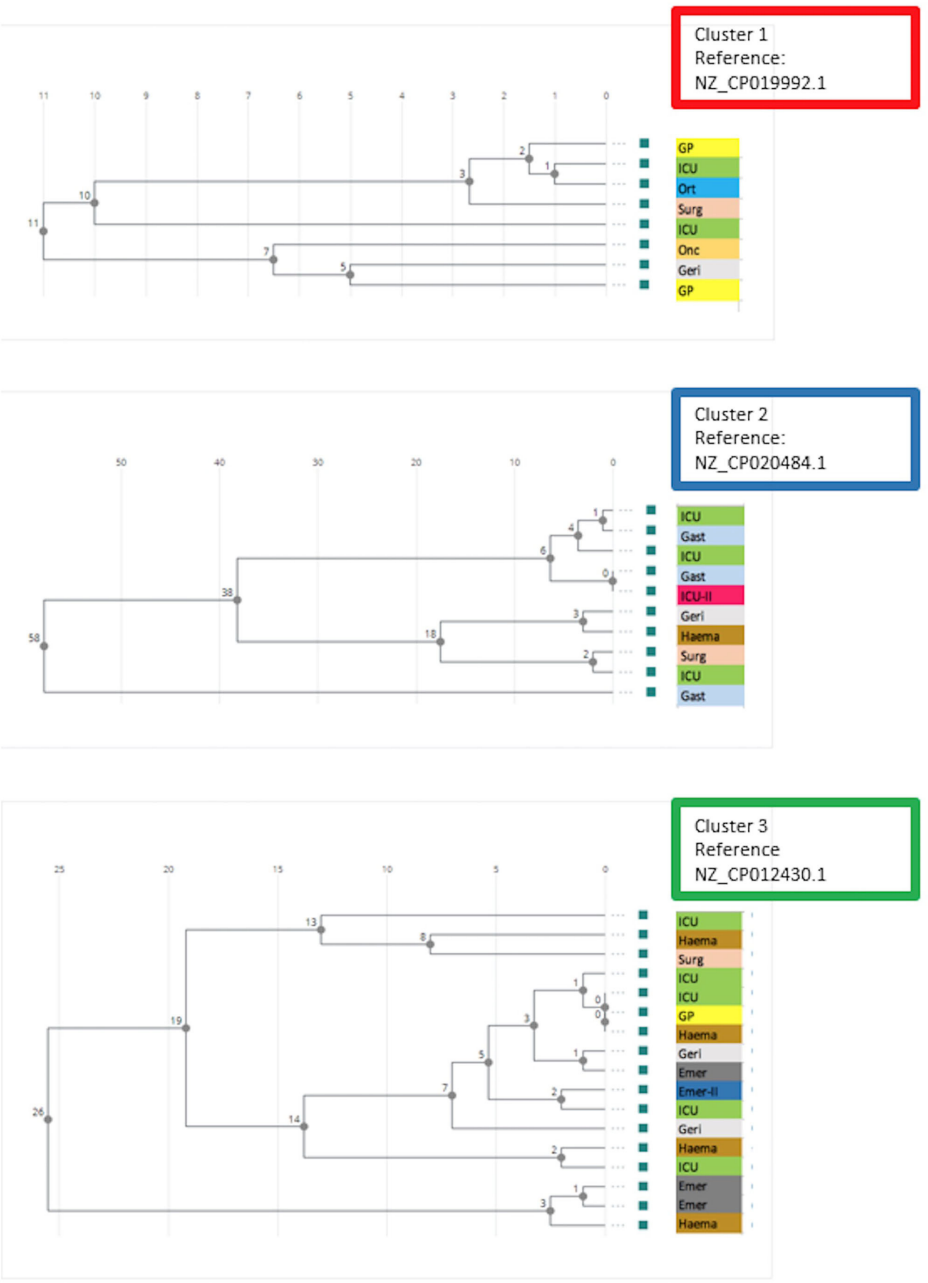
Phylogenetic trees based on SNPs in the core genome of *E. faecium* carrying *vanA* gene in the three clusters from Figure 1. Reference chromosome genome used for the analysis is indicated for each cluster. Location for sampling (General Practitioner or hospital department) is indicated for each isolate. ICU, Intensive Care Unit (hospital location 1); Ort, orthopedic surgery; Surg, abdominal surgery; Onc, oncology; Geri, geriatrics; GP, General Practitioner; Gast, gastroenterology; ICU-II, Intensive Care Unit at second hospital location; Haema, hematology; Emer, emergency; Emer-II, emergency at second location.

Three clusters were evident form the cgMLST based UPGMA dendrogram (Figure 1). Cluster 1 consisted of eight isolates of *vanA* gene positive *E. faecium* ST1421. All isolates in this cluster were from the second half of 2018 (Figure 2). The SNP analyses (Figure 3) showed few SNP differences between isolates. In addition to the *vanA* gene, the isolates all carried resistance genes to aminoglycosides [*aac(6'), ant(9')-la, aph(3')-IIIa*, macrolide-lincosamide-streptogramin B (MLS), *erm(A), erm(B), msr(C)*, and trimethoprim *dfr(G)*]. All resistance genes had more than 98.5% amino acid match and 100% length match compared to the reference sequence. Six isolates were cultured from patients at five hospital departments and two isolates were from patients consulting General Practitioners.

Cluster 2 included 10 ST203 isolates carrying *vanA* gene. The bacteria were cultured from first half of 2017 onwards (Figure 2). The isolates were cultured from patients at seven hospital departments. The isolates in Cluster 2 SNP analyses showed larger variation compared to the isolates in cluster 1 (Figure 3). All isolates in this cluster carried resistance genes to aminoglycosides [*aac(6'), ant(6')-la*, MLS *erm(B), msr(C)*, trimethoprim (*dfrG*), and tetracycline *tet(M), tet(U)*]. Except for one isolate with a length fraction of 94.3% of the reference sequences, all resistance genes had more than 98.5% amino acid identity and 100% length to the references. The *catA* gene, an effector of chloramphenicol resistance, was detected in seven of the isolates. The amino acid sequence and length were both 100% identical to the reference sequence.

Cluster 3 included 17 *vanA* gene positive isolates. Sixteen of the isolates were typed as ST80, while one was not typeable. MLST 2.0 (https://cge.cbs.dtu.dk/services/MLST/) also failed to type this isolate. The isolates in cluster 3 were cultured from 2016 onwards and showed numbers of SNP differences between those detected in Cluster 1 and Cluster 2. The isolates carried resistance genes to aminoglycosides [*aac(6'), aph(3')-IIIa*, MLS *erm(B), msr(C)*, and tetracyclines *tet(M)*]. With three exceptions both identity and fragment length were more than 98% of the reference sequences for all of these resistance genes. The *E. faecium* in this cluster were collected from six departments and one GP from 2016 onwards.

## Discussion

WGS offers a unique method for typing and monitoring healthcare associated bacteria such as enterococci carrying resistance genes to vancomycin. For the technique to be useful in clinical practice, the bioinformatic handling of sequence data should be easy and fast. Access to external sequencing facilities may provide high quality genome sequences without data analyses and interpretation. We used 1928 Diagnostics, a new online tool for bioinformatic analyses of transmissible bacteria, to review the genetic characteristics of *E. faecium* carrying genes encoding resistance to vancomycin at the hospital during a period of 5 years.

The genomes of the ST80 isolates were highly heterogenous, including isolates carrying *vanA* gene and isolates with *vanB* gene. One isolate of ST80 *E. faecium* carried both *vanA* and *vanB* genes as did 5% of vancomycin-resistant *E. faecium* in Denmark in 2018 (5). Heterogenous ST80 vancomycin-esistant *E. faecium* has been reported from Denmark (8). Recent studies of ST80 vancomycin-resistant *E. faecium* in Sweden showed comparable epidemiological discrimination by 1,928 Diagnostics cgMLST and SNP analyses (9).

Three major clusters of related isolates were evident. The clusters replaced each other over time as the most frequently isolated strain and isolates of the strains within each cluster continued to be detected afterwards. The Cluster 1 strain of *E. faecium* ST1421 carried the *vanA* gene without necessarily expressing resistance to vancomycin *in vitro* (10). This strain was frequently detected in Denmark in 2018 and 2019 (5). Cluster 2 and Cluster 3 also reflected the nation-wide occurrence of dominating strains of *E. faecium* carrying *vanA* gene during the 5 years (5). Genes encoding resistance to antibiotics other than vancomycin varied between clusters and were largely identical in isolates within each cluster. Knowledge on mechanisms of resistance to antimicrobials other than vancomycin may potentially contribute to treatment guidance and to tracking routes of transmission of antimicrobial resistance.

The detailed relatedness between isolates within each cluster was established by SNP analyses. No association between place of sampling and relatedness of isolates was evident. However, location of sampling does often not reflect place of transmission. It is noteworthy, that bacteria of all three clusters were isolated from patients referred to the same Intensive Care Unit and that 10 of the isolates were from patients admitted to this unit. This may be due to patient factors, use of broad-spectrum antimicrobials, extensive use of indwelling foreign body materials, and frequent sampling practice.

In conclusion, this retrospective study showed that a rapid and easy-to-use bioinformatic pipeline clearly separated isolates of *E. faecium* with resistance genes to vancomycin into clusters and sporadic strains and allowed detailed analyses of clusters of related isolates. Prospective studies should establish the usefulness of 1928 Diagnostics and similar systems for real time surveillance of vancomycin resistant *E. faecium* and other hospital-associated bacteria.

## Data Availability Statement

The original contributions presented in the study are publicly available. This data can be found at: https://www.ncbi.nlm.nih.gov/sra/?term=PRJNA767758.

## Author Contributions

MK, LM, and LA conceived and designed the study. MK, LM, CS, S-AS, AT, and LW analyzed the data. MK, LM, CS, S-AS, AT, LA, TA, and BK critically revised the manuscript. All authors read and approved the manuscript.

## Funding

The study was supported by a research grant from Odense University Hospital (Grant No.: 72-A3862).

## Conflict of Interest

The authors declare that the research was conducted in the absence of any commercial or financial relationships that could be construed as a potential conflict of interest.

## Publisher's Note

All claims expressed in this article are solely those of the authors and do not necessarily represent those of their affiliated organizations, or those of the publisher, the editors and the reviewers. Any product that may be evaluated in this article, or claim that may be made by its manufacturer, is not guaranteed or endorsed by the publisher.
